# Trimester-Specific Assessment of Diet Quality in a Sample of Canadian Pregnant Women

**DOI:** 10.3390/ijerph16030311

**Published:** 2019-01-24

**Authors:** Claudia Savard, Simone Lemieux, Élise Carbonneau, Véronique Provencher, Claudia Gagnon, Julie Robitaille, Anne-Sophie Morisset

**Affiliations:** 1 School of Nutrition, Laval University, Quebec City, QC G1V 0A6, Canada; claudia.savard.4@ulaval.ca (C.S.); simone.lemieux@fsaa.ulaval.ca (S.L.); elise.carbonneau.1@ulaval.ca (É.C.); veronique.provencher@fsaa.ulaval.ca (V.P.); julie.robitaille@fsaa.ulaval.ca (J.R.); 2 Endocrinology and Nephrology Unit, CHU of Québec-Université Laval Research Center, Quebec City, QC G1V 4G2, Canada; claudia.gagnon@crchudequebec.ulaval.ca; 3 Institute of Nutrition and Functional Foods, Laval University, Quebec City, QC G1V 0A6, Canada; 4 Department of Medicine, Laval University, Quebec City, QC G1V 0A6, Canada

**Keywords:** diet quality, Healthy Eating Index, pregnancy, prenatal nutrition

## Abstract

The present study aimed to (1) examine changes in diet quality throughout pregnancy and (2) identify maternal characteristics associated with trimester-specific diet quality. Pregnant women (*n* = 79) were recruited in their 1st trimester of pregnancy and completed, at each trimester, three web-based 24-hour dietary recalls, from which the Canadian Healthy Eating Index (HEI) was calculated. Physical activity, nutrition knowledge, and socio-demographic web-questionnaires were also completed. Although no variation in total HEI scores was observed across trimesters, we found an overall decrease in the following subscores: adequacy, total fruits and vegetables, unsaturated fats and saturated fats (*p* < 0.05). In the 1st trimester, overweight and obese pregnant women had a lower diet quality in comparison with normal-weight and underweight women (HEI scores: 63.1 ± 11.9 vs. 68.0 ± 9.3; *p* = 0.04). In the 3rd trimester, women younger than 28 years old, with no university degree, poorer nutrition knowledge and who reside in an urban setting, had a lower diet quality (*p* < 0.05). In conclusion, less educated, younger women who reside in an urban setting may be at a higher risk of poor diet quality in late pregnancy and could benefit from public health programs.

## 1. Introduction

Initiating and maintaining healthy eating behaviours is essential during pregnancy since poor maternal nutrition can adversely affect both the mother and her future child [1,2,3]. In the past, prenatal nutritional epidemiology was primarily concerned with the impact of malnutrition and nutritional deficiencies [4,5,6,7,8], but current literature is now increasingly interested in the overall quality of the maternal diet [9]. Combined assessment of maternal dietary intake and global diet quality would allow for (1) the detection of nutritional excesses and deficiencies, and (2) the identification of dietary patterns associated with adverse pregnancy outcomes [9]. For these reasons, various dietary patterns and diet quality indexes, such as the Healthy Eating Index (HEI) [10,11,12] were developed. Since then, greater diet quality during pregnancy has been associated with positive pregnancy outcomes [13]. A recent meta-analysis reported that a diet rich in fruits, vegetables, whole grains, and fish combined with lower intakes of red and/or processed meats and high-fat dairy products was associated with a lower risk of gestational diabetes [14]. Moreover, poorer diet quality during pregnancy has been associated with birthweight and neonatal adiposity [13,15]. However, in these studies, diet quality was not assessed at each trimester of pregnancy. It is important to address this gap in the literature, since diet during pregnancy may change and have different implications depending on the trimester during which diet is assessed [16,17]. In fact, during organogenesis, dietary intakes are more likely to play a role in the development of organs and systems, while during the fetal period, the diet would rather influence the growth and weight gain of the fetus as well as the mother’s gestational weight gain [16,17,18]. For example, folic acid supplementation is recommended before and during early pregnancy in order to reduce the risk of neural tube defects [19]. However, little is known about the role of folic acid during the 2nd and 3rd trimesters, i.e., the fetal period, and a study by Wang et al. recently reported an association between folic acid supplementation after the 1st trimester and the risk of large-for-gestational-age birth [20].

According to national guidelines, pregnant women should increase their daily energy intake progressively, i.e., in the 2nd and 3rd trimesters, in order to account for the high metabolic demand related to the fetus’ growth [21,22]. In Canada, pregnant women are advised to eat one to three additional servings from any of the four food groups (fruits and vegetables, grain products, milk and alternatives (yogurt, cheese, soy milk, almond milk, etc.), as well as meat and alternatives (red meat, fish, poultry, grains, nuts, legumes, etc.)) of Canada’s Food Guide (CFG), in order to increase their daily energy intake in the 2nd and 3rd trimesters [21]. 

Numerous studies have investigated pregnant women’s dietary intakes from a quantitative point of view, i.e., by the assessment of specific or various nutrient intakes [23,24,25,26,27]. However, very few studies have investigated overall diet quality during pregnancy and its determinants. A previous Canadian study by Nash et al. identified immigration status, parity, physical activity, smoking habits, anxiety, marital status, and social support as variables that could significantly impact maternal diet quality [28]. The authors concluded that further research was necessary to investigate potential determinants of diet quality. Although Nash et al. identified determinants of overall diet quality during pregnancy, they did not examine trimester-specific determinants of diet quality [28].

To our knowledge, diet quality was not assessed prospectively among Canadian pregnant women and trimester-specific determinants of diet quality need to be further investigated. To address this gap in the literature, our study aimed (1) to examine changes in diet quality throughout pregnancy using the HEI and adherence to CFG and (2) to identify maternal characteristics associated with trimester-specific diet quality.

## 2. Materials and Methods 

### 2.1. Study Population

Eighty-six (86) pregnant women were recruited at the CHU de Québec—Université Laval (Québec City, Canada) to participate in the ANGE project (*Apports Nutritionnels Durant la GrossessE*). The study protocol has been previously described [29]. Briefly, women were included if they were at least 18 years of age, had a gestational age less than 11 weeks and were able to communicate in French. Women were excluded if they had an inflammatory or autoimmune disease and if they planned to deliver in birthing centers or hospital centers other than the CHU de Québec-Université Laval. The ANGE project was approved by the CHU de Québec—Université Laval Research Center’s Ethical Committee (Reference number: 2016–2866) and participants gave their informed written consent at their first study visit to the research center (baseline visit). Seven women were lost at follow-up due to miscarriage (*n* = 3) or inability to devote time to the study (*n* = 4). Therefore, our final analyses include 79 pregnant women.

### 2.2. Nutritional Sata Collection

Each participant was asked to complete a total of 9 web-based 24-h dietary recalls; 3 in the 1st trimester (range: 8.4–14.0 weeks), 3 in the 2nd trimester (range: 19.3–28.3 weeks), and 3 in the 3rd trimester (range: 31.9–37.7 weeks). At each trimester, dietary recalls were completed on two weekdays and one weekend day. The dietary recalls were completed using the R24W (*Rappel de 24h Web*) platform. The R24W has been previously described and was recently validated in pregnant women and in the general population [29,30,31]. Briefly, the R24W uses a sequence of questions adapted from the United States Department of Agriculture (USDA) Automated Multiple Pass Method (AMPM) [32]. The application sends automatic emails on randomly chosen dates to remind the participants to complete the recall. The database includes 2865 food items that are linked to the Canadian Nutrient File [33] to enable automatic extraction of nutrient values. Pictures depicting multiple portion sizes with corresponding units or volume are available for more than 80% of all food items. After selecting a food item, participants chose the picture that best represented the amount of food eaten. All food items were automatically coded using the 2015 version of the Canadian Nutrient File [33]. The number of servings for each CFG group was calculated automatically by the R24W platform [30,34]. To compare the number of servings of our study sample with CFG’s recommendations, we used the recommended range of servings for non-pregnant adult women (for comparisons with 1st trimester servings), to which we added 2 and 3 additional servings, for comparisons with the 2nd and 3rd trimesters reported servings, respectively.

### 2.3. Healthy Eating Index

Diet quality was assessed using the 2007 version of the Canadian HEI (C-HEI) [35], which is automatically calculated by the R24W platform. The C-HEI is an adaptation of the HEI developed by Kennedy et al. [11] and its assessment relies on the number of servings reported by the participant, according to age and sex, as specified in the latest version of the CFG, which was published in 2007 [34,35]. Briefly, the Canadian HEI is comprised of eight *adequacy* components and three *moderation* components (see Table S1) [35]. For each component, points between 0 and the potential maximum score (5, 10, or 20) are assigned. An individual receives no points if they have failed to meet the criterion altogether, the maximum score is assigned if the criterion is met perfectly; and a proportional score is assigned if the reported intake falls between the two extremes. Scores are then added up for a maximum of 100 points, representing a perfect adherence to CFG. In this study, complex meals were firstly decomposed into their main components. All food items and main components of complex dishes were then translated into CFG servings, from which HEI sub-scores are calculated [35]. Total HEI scores of each day (3 days per trimester) were computed and averaged by the R24W platform. The detailed information regarding the range of scores and scoring criteria is presented in Table S1. The HEI has been used by various authors to assess diet quality among pregnant women [36,37,38].

### 2.4. Supplement Use 

A Web questionnaire administered at each trimester was completed by all participants in order to collect information on supplement use. Participants had to identify their supplement (e.g., brand name, type of supplement, specific nutrient, etc.), provide its drug identification number, its measurement unit (e.g., tablet, drop, gram, milliliter, etc.), its dose and the frequency at which the reported dose was taken (e.g., once a day, twice a week, etc.). We used the Health Canada Licensed Natural Health Product Database [39] as well as the companies’ product labels and websites in order to collect the nutritional information of all supplements entered by participants. If information was missing or incomplete for any of the supplements’ characteristics, a research assistant contacted the participant to obtain the missing information. Supplement use was assessed by compiling the types of supplement reported (multivitamin or single nutrient) and the number of users for each type of supplement.

### 2.5. Nutrition Knowledge

During their 1st trimester of pregnancy, participants completed a web-based self-administered questionnaire that assessed their general nutrition knowledge. The development of this questionnaire has been previously described and was validated among French-Canadian adults [40]. Questions are mainly related to the knowledge of CFG (e.g., number of servings recommended and food items included in each food group), but some also assess general nutrition knowledge (e.g., agree/disagree ‘All spices have a high sodium (salt) content’). Total score ranges from 0 to 13.5, 13.5 being the highest value given for knowledge of nutrition. In our analyses, participants were classified according to the median score of our sample. Women scoring equal to or above the median were considered as having a better nutrition knowledge.

### 2.6. Nausea, Vomiting and Food Preferences During Pregnancy

At each trimester, a web-based self-administered questionnaire regarding nauseous symptoms as well as food cravings and aversions was completed. If pregnant women reported having experienced nausea and/or vomiting, they were asked to specify how frequently these symptoms occurred. Finally, women were asked if they had experienced any food cravings and/or aversions since they became pregnant and if so, which foods were craved and/or disliked. All food items listed were analyzed by a registered dietitian and then divided into 9 food categories (see Results section). In our analyses, women were classified as having (yes) or not (no) reported nausea, vomiting, food cravings, and/or food aversions.

### 2.7. Other Variables

Pre-pregnancy BMI was calculated using self-reported pre-pregnancy weight and the height measured at the first study visit to the research center (baseline visit). Gestational age was confirmed by a dating ultrasound conducted at the CHU de Québec—Université Laval in the 1st trimester. Participants completed the validated web-based French version of the Pregnancy Physical Activity Questionnaire (PPAQ) [41,42] at each trimester. Physical activity level was determined by ranking participants according to the total amount of time they engaged in moderate and high-intensity activities (minutes/day). Women were either categorized as active (≥30 min/day) or less active (<30 min/day). Finally, participants completed a web-based self-administered questionnaire to collect information on economic and socio-demographic characteristics such as education level, annual income, and living environment. In this questionnaire, participants were asked whether they lived in an urban (in the city), suburban or rural (countryside) setting.

### 2.8. Statistical Analyses

Descriptive analyses were conducted to characterize the study population. Age was categorized according to the Canadian average age at first birth (28 years old) [43]. Pre-pregnancy BMI was divided in 2 categories to compare underweight/normal-weight women vs. overweight/obese women (<25 kg/m^2^ and ≥25 kg/m^2^). At each trimester, mean HEI scores (total and sub-scores) as well as the mean number of CFG servings were calculated from the three recalls. Repeated measures analyses of variance (MANOVA) were performed to assess variations, across trimesters, in diet quality, using the two indicators described above (i.e., HEI and CFG servings). Pairwise comparisons were performed for components for which a significant variance was observed across trimesters. Further repeated measures analyses of variance were performed for total HEI score of subsamples of participants in order to compare variations in diet quality according to different maternal characteristics (i.e., age, primiparity, pre-pregnancy BMI, physical activity, multivitamin use, income, education, nutrition knowledge, living environment, reported food cravings/aversions, and nauseous symptoms). One-way analyses of variance (ANOVA) were used to compare trimester-specific HEI scores according to the maternal characteristics previously listed. Finally, stepwise regressions were performed with the predictor variables on trimester-specific HEI score. A cut point of *p* < 0.25 was used in the stepwise regression, in order to identify which variables could be entered in the model. The same maternal characteristics listed above were tested in the Stepwise procedure. For all statistical analyses, differences were considered to be statistically significant at *p* ≤ 0.05 and *p* ≤ 0.10 were considered as trends. Variables that were not normally distributed (Pre-pregnancy BMI, 1st trimester HEI score and physical activity in all trimesters) were transformed (Log_10_ or Boxcox) in order to perform analyses that require a normal distribution (Stepwise regression). All statistical analyses were performed using JMP version 13.2.1 (SAS Institute Inc., Cary, NC, USA).

## 3. Results

### 3.1. Participants’ Characteristics 

Participants’ characteristics are presented in Table 1. Final analyses include 79 pregnant women recruited at 9.3 ± 0.7 weeks of pregnancy, aged 32.1 ± 3.7 years old on average and with a mean pre-pregnancy BMI of 25.7 ± 5.8 kg/m^2^. Seventy-four (93.7%) of the women filled all nine dietary recalls. The five other women (6.3%) completed eight of the nine required R24W (data not shown). More specifically, two women missed one recall in the 1st trimester, two different women missed one recall in the 2nd trimester and another woman missed one recall in the 3rd trimester. Only two women (2.5%) were categorized as being underweight. The majority of participants were Caucasian (97.5%), multiparous (64.6%), resided in an urban/suburban area (89.8%), were university degree holders (78.5%), and had an annual household income of 80,000 Canadian dollars or more (63.3%). Physical activity decreased throughout pregnancy (*p* = 0.0024) and participants scored a mean of 9.6 ± 1.6 (range 5.1–12.8) out of 13.5 on the nutrition knowledge questionnaire. Most women (88.6%) reported nausea in the 1st trimester of pregnancy. Significantly fewer women reported nauseous symptoms in the 2nd and 3rd trimesters (32.9% and 20.3%, respectively vs. 88.6% in the 1st trimester; *p*-value of MANOVA < 0.0001) and fewer participants reported having experienced vomiting throughout their pregnancy (31.7%, 19.0%, and 2.5% in the 1st, 2nd, and 3rd trimesters, respectively; *p*-value of MANOVA < 0.0001). Most women reported food cravings (55.7%) and aversions (63.3%) in their 1st trimester and these proportions decreased across trimesters (*p* = 0.0095 for cravings and *p* = 0.0001 for aversions; Table 1). Additional details on the frequency of nausea and vomiting symptoms and information regarding categories of foods craved and disliked are presented in Tables S2 and S3. 

### 3.2. Supplement Use

Data on supplement use is presented in Table S4. A majority of women reported using prenatal multivitamins (86.1%, 84.8%, and 78.5% in the 1st, 2nd, and 3rd trimesters). Folic acid supplements were the most commonly reported single-nutrient supplements (data not shown). Only a small proportion (<10%) of women reported taking vitamin D, iron, and omega-3 as single-nutrient supplements throughout pregnancy (Table S4). 

### 3.3. Adherence to Canada’s Food Guide

Table 2 shows the adherence to CFG recommendations for each food group and subgroup throughout pregnancy. Average fruit and vegetable servings (6.4 ± 2.2 in the 1st, 6.1 ± 2.6 in the 2nd, and 5.8 ± 2.4 in the 3rd; *p* = 0.094) decreased throughout pregnancy and did not meet the minimum recommended number of servings of seven. Women were, on average, within the recommended ranges for the three other food groups. An increase in the number of servings throughout pregnancy was observed only for the ‘milk and alternatives’ food group. (2.5 ± 1.1, 2.7 ± 1.4, and 3.0 ± 1.4 in the 1st, 2nd and 3rd trimesters, respectively; *p* = 0.002). 

### 3.4. Diet Quality throughout Pregnancy

Average HEI scores (total and subscores) are presented in Table 3. Total HEI scores did not significantly vary throughout pregnancy (65.8 ± 10.8 in the 1st, 65.0 ± 12.0 in the 2nd, and 62.9 ± 12.6 in the 3rd trimester; *p* = 0.075). In contrast, the adequacy sub-score significantly decreased across trimesters (47.2 ± 7.4, 46.4 ± 7.7, 44.7 ± 8.2; in the 1st, 2nd and 3rd trimesters, respectively; *p* = 0.016). Among the adequacy component, total vegetables/fruits as well as unsaturated fats sub-scores significantly decreased throughout pregnancy (*p* < 0.05). Saturated fats sub-scores (part of the moderation component) also decreased throughout pregnancy (*p* < 0.05). 

Pairwise comparisons for adequacy and unsaturated fats scores showed a significant decrease between the 1st and 3rd trimesters (*p* < 0.05) and between the 2nd and 3rd trimesters (*p* < 0.05), respectively. Total fruits and vegetables scores decreased between the 1st and 2nd trimesters (*p* < 0.05) and between the 1st and 3rd trimesters (*p* < 0.05). Finally, saturated fats scores decreased only between the 1st and 3rd trimesters (*p* < 0.05). When variations in diet quality were analyzed by maternal characteristics, the stability across trimesters previously observed was maintained, with the exception of a significant decrease in HEI scores in women who were less active in the 1st and 3rd trimesters, were living in an urban setting and who scored lower on the nutrition knowledge questionnaire (*p*-value of MANOVA < 0.05; data not shown).

### 3.5. Trimester-Specific Diet Quality

**Figure 1:**
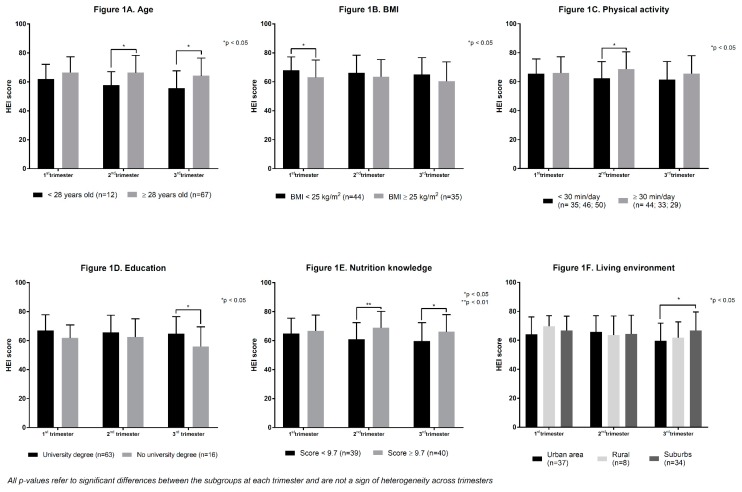
Trimester-specific diet quality according to maternal characteristics: (**A**) Age; (**B**) BMI; (**C**) Physical activity; (**D**) Education; (**E**) Nutrition knowledge; (**F**) Living environment.

Figure 1 shows differences in trimester-specific HEI scores according to maternal characteristics. Higher pre-pregnancy BMI, lower maternal age, lower physical activity, lower education level, poorer nutrition knowledge and an urban living environment were all associated with poorer diet quality. However, as shown in Figure 1, the maternal characteristics associated with poorer diet quality varied across trimesters. No significant differences were observed in HEI scores of primiparous vs multiparous women and women who did or did not report nausea, food cravings, and aversions (data not shown). Women who did not report vomiting in the 1st trimester tended to have a lower HEI score compared to women that did report vomiting symptoms (*p* = 0.098; data not shown). Moreover, women that did not take a multivitamin in the 1st trimester tended to have a lower HEI score compared to women who did take one (*p* = 0.0531; data not shown).

Table 4 shows results of the stepwise regression of trimester-specific HEI to identify predictor variables. In the 1st trimester, reported vomiting (*r*^2^ = 0.10; *β* = 0.34; *p* = 0.003), multivitamin use (*r*^2^ = 0.05; *β* = 0.23; *p* = 0.042) and pre-pregnancy BMI (*r*^2^ = 0.04; *β* = −0.22; *p* = 0.047) were identified as significant predictors of total HEI score, in a model that also included nutrition knowledge, living environment and education level. In the 2nd trimester, only physical activity (*r*^2^ = 0.07; *β* = 0.28; *p* = 0.012) was a significant predictor of total HEI score, in a model that also included nutrition knowledge, reported vomiting and food cravings. Finally, in the 3rd trimester, only nutrition knowledge (*r*^2^ = 0.07; *β* = 0.26; *p* = 0.017) was a significant predictor of total HEI score, in a model also including pre-pregnancy BMI, living environment and education level.

## 4. Discussion

Our research team recently published a trimester specific assessment of nutrient intakes in comparison with national dietary guidelines, in the same sample of pregnant women, but that paper did not assess overall diet quality during pregnancy [23]. This paper is therefore, to our knowledge, the first to assess trimester-specific diet quality and adherence to CFG in Canadian pregnant women, as well as identifying maternal characteristics associated with diet quality throughout pregnancy. In all trimesters, intake of fruits and vegetables did not meet the CFG recommended number of servings and decreased throughout pregnancy. In contrast, ‘milk and alternatives’ intake increased significantly across trimesters. Overall, total HEI score remained stable throughout pregnancy. Sub-scores of total fruits/vegetables and unsaturated fats significantly decreased throughout pregnancy. In the 1st trimester, overweight and obese women had poorer diet quality compared with normal-weight and underweight women. Later in pregnancy, women younger than 28 years old that were less active, residing in an urban setting, with no university degree, and poorer nutrition knowledge had lower HEI scores than older, more active, educated women with better nutrition knowledge who lived in the suburbs. The best predictors of poorer diet quality were (1) unreported vomiting, lack of multivitamin use, and higher pre-pregnancy BMI; (2) lower physical activity level; and (3) poorer nutrition knowledge, for the 1st, 2nd, and 3rd trimesters, respectively. 

An overall decrease in maternal diet quality was observed by Moran et al. [36] in a study that assessed the HEI score of 301 overweight and obese Australian pregnant women at each trimester and at 4 months post-partum. Similarly, it has been suggested that women may change their diet after learning that they are pregnant, which could be explained by the fact that pregnancy itself is associated with a higher motivation to adopt healthy eating habits [44,45,46]. In the present study, since we did not assess pre-pregnancy diet, it is not possible to evaluate any diet changes between pre-pregnancy and early pregnancy. Nevertheless, a prospective study by Skreden et al. [47] observed an increase in the proportion of Norwegian pregnant women consuming fruits and vegetables daily or more frequently from pre-pregnancy to early (9–20 weeks) pregnancy. It could then be hypothesized that a decrease in motivation, from early to late pregnancy, could occur in certain women and therefore be associated with a decrease in overall diet quality as the pregnancy progresses. In our study, this could partly explain the fact that the total fruits/vegetables, unsaturated and saturated fats sub-scores were higher in the 1st trimester compared with the 2nd and 3rd trimesters. However, changes in the motivation to maintain healthy eating habits during pregnancy should be further investigated in association with other determinants of diet quality. In addition, it would be interesting to assess diet quality after birth and/or during the breastfeeding period, in order to examine whether and how pregnancy impacts a woman’s diet in the long-term.

In our study, overweight and obese pregnant women had, in the 1st trimester only, lower HEI scores compared to normal-weight and underweight women. Similarly, Shin et al. [48] assessed the HEI score of 795 American pregnant women once (various trimesters) and found an inverse association between pre-pregnancy BMI and maternal diet quality as well as nutritional biomarkers. Tsigga et al. [38] also found, using three dietary recalls once during pregnancy (trimester unspecified) among 100 Greek pregnant women, that overweight and obese women had a lower HEI score than their counterparts. Following these observations, it is relevant to mention that a poorer diet quality prior to pregnancy might contribute to a higher pre-pregnancy BMI. Consequently, it would be considered logical that, in our study, obese and overweight women had lower 1st trimester HEI scores. Once again, since we do not have any data regarding pre-pregnancy diet, we cannot verify these hypotheses. Nevertheless, our results combined with those of other authors suggest that women with a higher pre-pregnancy BMI are at higher risk of poorer diet quality during and after pregnancy and should therefore be monitored early on in pregnancy. However, in our study, pre-pregnancy BMI was not a significant predictor of diet quality in the 2nd and 3rd trimesters. It could then be hypothesized that diet quality is influenced by different factors in late pregnancy in comparison with early pregnancy. Moreover, given that overweight and obese pregnant women are at higher risk of gestational diabetes, hypertension disorders, and numerous other adverse pregnancy outcomes; their diet, especially in early pregnancy, should be monitored closely [49,50,51]. 

In the 2nd trimester, diet quality was lower in women younger than 28 years old, who were less active and had poorer nutrition knowledge. Differences in HEI scores related to age and nutrition knowledge persisted in the 3rd trimester where, additionally, women lacking a university degree, living in an urban setting (vs. the suburbs) also had lower diet quality. Similar differences were observed by Nash et al. [28], Rifas-Shiman et al. [52], Laraia et al. [53], who found that women who were younger, less educated, less physically active, and who lived within 500 m of fast-food restaurants and convenience stores, had lower diet quality during pregnancy. Doyle et al. [54] observed similar associations and highlighted the need for more studies assessing diet quality in combination with environmental factors, as well as a consideration for pregnancy to be taken into account as a determinant factor itself. Although our results are in line with the literature, it is still important to further investigate diet quality during pregnancy in association with maternal, environmental, behavioural, and sociodemographic characteristics, in order to identify pregnant women at higher risk of nutritional inadequacies that could benefit the most from a nutritional intervention. Furthermore, in our study, maternal characteristics associated with poorer diet quality varied across trimesters, which suggest that diet quality may not be influenced by the same factors throughout pregnancy. Moreover, although some factors may help identify the women that are at higher risk of poorer diet quality, some of them are not necessarily modifiable (e.g., education level, living environment, age, etc.). Therefore, other modifiable factors (e.g., nutritional knowledge, physical activity level, etc.) could be targeted for nutritional interventions during pregnancy.

Stepwise regression analyses of HEI scores identified different predictors of diet quality depending on the trimesters. We found that the best predictors of poorer diet quality were: (1) the fact that women did not experience vomiting, lack of multivitamin use, and a higher pre-pregnancy BMI; (2) lower physical activity; and (3) poorer nutrition knowledge, for the 1st, 2nd, and 3rd trimesters, respectively. In comparison, Nash et al. [28] observed in their sample of 2282 Canadian pregnant women that the best determinants of greater diet quality were: being an immigrant residing in Canada for ≤5 years, marriage and multiparity, physical activity, lower anxiety levels, and greater social support from family. Apart from physical activity, our results differ from those of Nash et al. [28], possibly due to our small and homogenous sample. Support from family, anxiety levels, and marital status were not assessed in our study. Moreover, since only two of our participants were immigrants, we did not include this variable in our stepwise procedures. Nevertheless, the predictors identified in our study only explained a small proportion of the variability in diet quality (Total *r*^2^ values of the models varied between 0.14 and 0.27). As previously mentioned, there are many factors that could contribute to maternal diet quality that were not included in our study [28,55,56]. Future studies should consider including environmental, psychosocial, socio-demographical as well as biological variables in multivariate models in order to obtain a more global picture of which variables best predict diet quality during pregnancy. In addition, as the best predictors of diet quality differed according to trimesters, it is important that diet quality be assessed prospectively, at each trimester.

Two major strengths of this study were its prospective design and its early enrollment of participants, allowing us to examine changes in diet quality throughout pregnancy. Moreover, the use of a validated web-based 24 h dietary recall generated detailed information on dietary intakes thus allowing a precise assessment of diet quality as well as the evaluation of adherence to CFG. Our study has some limitations, namely regarding the small size and the homogeneity of our sample; since most of the pregnant women who were enrolled were Caucasians and of a higher socioeconomic status. Still, even though our sample was highly educated and reported a high annual income, average intake of fruits and vegetables did not meet CFG’s recommendations. We can hypothesize that less educated and lower-income women may be at a greater risk of poor diet quality and nutritional inadequacies. Our small sample size might also have attenuated the statistical significance of our results. Still, diet quality significantly differed between some subgroups. The variations we observed in some HEI sub-scores are possibly continuous throughout pregnancy, which we cannot confirm. For a better estimate and a more continuous evaluation of diet quality, additional R24Ws would have been needed. Yet, asking our participants to recall and report their dietary intakes for more than three days per trimester could have worsened compliance, participation rate, and potentially altered our results. In addition, it has been previously stated that three days are representative of a pregnant woman’s usual diet [2,22]. Finally, our study did not assess important psychosocial factors like stress and anxiety that could have impacted on diet quality during pregnancy. Interactions between such factors and diet quality should be further investigated.

## 5. Conclusions

Overall diet quality did not vary throughout pregnancy, but sub-scores related to fruits and vegetables, unsaturated and saturated fats significantly decreased across trimesters. Although pregnancy is known to be a key period during which pregnant women are motivated to adopt healthy behaviors, it is possible that motivation decreases as pregnancy progresses, making it difficult for women to maintain the quality of their diet. Women who were overweight and obese had poorer diet quality in early pregnancy compared to normal-weight and underweight women. Our study also showed that women under 28 years of age, less educated, less active, and who live in an urban setting may be at a higher risk of poorer diet quality in late pregnancy. These women may benefit the most from interventions centered on healthy eating behaviors. Furthermore, variables associated with poorer diet quality varied across trimesters, which suggests that diet quality may not be influenced by the same factors throughout pregnancy. Hence, highlighting the need to monitor diet quality at various points during pregnancy. Future studies should assess trimester-specific diet quality in association with environmental, psychosocial, biological, as well as socio-demographical factors.

## Figures and Tables

**Table 1:** Participants’ characteristics (*n* = 79).

Variables	Mean ± SD or *N* (%)		
	Baseline (1st Trimester)	2nd Trimester	3rd Trimester
Age (years)	32.1 ± 3.7	-	-
Weeks of gestation at baseline (weeks)	9.3 ± 0.7	-	-
Primiparous	28 (35.4)	-	-
BMI (kg/m^2^)	25.7 ± 5.8	-	-
Underweight	2 (2.5)	-	-
Normal weight	43 (54.4)	-	-
Overweight	19 (24.1)	-	-
Obese	15 (19.0)	-	-
Ethnicity-Caucasian ^1^	77 (97.5)	-	-
Education			
High school	4 (5.0)	-	-
College	13 (16.5)	-	-
University	62 (78.5)	-	-
Household income			
<60,000 $	15 (19.0)	-	-
60,000–79,999 $	13 (16.5)	-	-
80,000–99,999 $	17 (21.5)	-	-
>100,000 $	33 (41.8)	-	-
Income missing	1 (1.2)	-	-
Living environment			
Urban	37 (46.8)	-	-
Rural	8 (10.1)	-	-
Suburbs	34 (43.0)	-	-
General nutrition knowledge (total score) ^2^	9.6 ± 1.6	-	-
Physical activity level (min/day) ^3^	60.5 ± 59.6	45.9 ± 51.1	35.2 ± 41.5
Reported nausea (yes)	70 (88.6)	26 (32.9)	16 (20.3)
Reported vomiting (yes)	25 (31.7)	15 (19.0)	2 (2.5)
Food cravings (yes)	44 (55.7)	33 (41.8)	25 (31.7)
Food aversions (yes)	50 (63.3)	28 (35.4)	17 (21.5)

^1^ Other ethnicities were: Middle-Eastern (*n* = 1) and Venezuelan (*n* = 1); ^2^ Sum of moderate- and vigorous-intensity physical activity assessed by the PPAQ; ^3^ Total score ranges from 0 to 13.5, assessed by the Nutrition Knowledge Questionnaire developed by Bradette-Laplante et al. (2017) [40].

**Table 2:** Servings of 2007 Canada’s Food Guide food groups and subgroups throughout pregnancy.

Food Groups	Number of Servings/Day (or Other) ^1^						*p*-Value *
	1st Trimester		2nd Trimester		3rd Trimester		
	Recommended Range ^2^	Mean ± SD	Recommended Range ^3^	Mean ± SD	Recommended Range ^4^	Mean ± SD	
Fruits and vegetables	7–8	6.4 ± 2.2	7–10	6.1 ± 2.6	7–11	5.8 ± 2.4	0.094
Whole fruits	-	2.0 ± 1.1	-	2.0 ± 1.3	-	2.0 ± 1.2	0.875
Green and orange vegetables	2	1.3 ± 0.8	2–4	1.2 ± 0.9	2–5	1.1 ± 0.8	0.321
Grain products	6–7	6.1 ± 1.8	6–9	5.9 ± 1.8	6–10	5.7 ± 2.0	0.206
Whole grain products	3	1.5 ± 1.3	3–5	1.5 ± 1.3	3–6	1.6 ± 1.4	0.868
Milk and alternatives	2	2.5 ± 1.1	2–4	2.7 ± 1.4	2–5	3.0 ± 1.4	**0.002**
Meat and alternatives	2	2.2 ± 0.9	2–4	2.2 ± 0.8	2–5	2.1 ± 0.8	0.740
Saturated fats (% daily EI)	-	12.8 ± 2.1 22.2 ± 9.6	-	13.2 ± 2.9	-	13.5 ± 2.5	**0.047**
Other foods (% daily EI)	-		-	22.0 ± 9.5	-	22.1 ± 10.3	0.969

^1^ For specific information regarding serving sizes of each food group, please refer to CFG [34]; ^2^ Recommendations for non-pregnant adult women; ^3^ 1st trimester recommendations + 2 portions; ^4^ 1st trimester recommendations +3 portions; EI: Energy intake; * *p*-value of the repeated measures analyses of variance performed across trimesters, bold characters indicate significance of the analysis.

**Table 3:** Healthy Eating Index total and subscores throughout pregnancy.

HEI	1st Trimester	2nd Trimester	3rd Trimester	*p*-Value *
Total	65.8 ± 10.8	65.0 ± 12.0	62.9 ± 12.6	0.075
Adequacy ^†^	47.2 ± 7.4	46.4 ± 7.7	44.7 ± 8.2	**0.016**
Total vegetables and fruits	8.3 ± 2.0	7.7 ± 2.1	7.7 ± 2.2	**0.018**
Whole fruits	4.3 ± 1.5	4.1 ± 1.4	4.3 ± 1.4	0.778
Dark green and orange vegetables	3.5 ± 1.6	3.4 ± 1.6	3.0 ± 1.6	0.056
Total grain products	4.5 ± 0.7	4.4 ± 0.8	4.2 ± 0.9	0.123
Whole grains	2.4 ± 1.8	2.3 ± 1.7	2.4 ± 1.9	0.893
Milk and alternatives	8.9 ± 2.0	8.9 ± 2.1	9.1 ± 1.9	0.685
Meat and alternatives	8.7 ± 2.0	8.9 ± 1.9	8.6 ± 2.2	0.566
Unsaturated fats	6.6 ± 3.0	6.6 ± 3.4	5.4 ± 3.4	**0.008**
Moderation ^‡^	18.6 ± 7.2	18.6 ± 7.9	18.2 ± 8.1	0.894
Saturated fats	4.0 ± 2.7	3.6 ± 3.1	3.1 ± 2.8	**0.039**
Sodium	4.4 ± 2.6	4.7 ± 2.8	5.0 ± 2.7	0.173
Other foods	10.2 ± 5.1	10.3 ± 5.2	10.2 ± 5.7	0.970

* *p*-value of the repeated measures analyses of variance performed across trimesters, bold characters indicate significance of the analysis. ^†^ for adequacy components, 0 points for minimum intake or less, 5, 10 or 20 for maximum intake or more, and proportional for amounts between minimum and maximum. ^‡^ for moderation components, 10 or 20 points for minimum intake or less, 0 points for maximum intake or more, and proportional for amounts between minimum and maximum.

**Table 4:** Stepwise regression analyses of trimester-specific HEI scores.^a.^

Maternal Characteristics	1st Trimester			2nd Trimester			3rd Trimester		
	*r*^2^ × 100	B ^b^	*p*-Value	*r*^2^ × 100	*β*	*p*-Value	*r*^2^ × 100	β	*p*-Value
Vomiting (yes/no)	**10.0**	**0.34**	**0.003**	1.9	−0.14	NS	-	-	-
Multivitamin use (yes/no)	**4.5**	**0.23**	**0.042**	-	-	-	-	-	-
Pre-pregnancy BMI (kg/m^2^)	**4.2**	**−0.22**	**0.047**	-	-	-	4.5	−0.20	NS
Nutrition knowledge (score)	3.5	0.21	NS	3.5	0.19	NS	**6.8**	**0.26**	**0.017**
Living environment ^c^	3.3	0.32	NS	-	-	-	6.7	0.16	NS
University degree (yes/no)	1.7	0.14	NS	-	-	-	2.6	0.16	NS
Physical activity (minutes/day)	-	-	-	**7.4**	**0.28**	**0.012**	-	-	-
Food cravings (yes/no)	-	-	-	1.6	−0.13	NS	-	-	-
Total	27.3			14.4			20.6		

Bold indicates significance of the variable. ^a^ *β* coefficients, r^2^ × 100 and p-values are not shown for variables that were not included in the regression model (*p* > 0.25) following the stepwise procedure. ^b^ *β* coefficients of the 1st trimester analyses represent the degree of change in BoxCox transformed HEI score, since this variable was not normally distributed in the 1st trimester. ^c^ Living environment refers to living in the suburbs or in a rural setting, as opposed to an urban setting.

## Supplementary Materials

The following are available online at http://www.mdpi.com/1660-4601/16/3/311/s1, Table S1: Components of Canadian adaptation of Healthy Eating Index, range of scores and scoring criteria, Table S2: Frequency of nausea and vomiting among participants, Table S3: Food cravings and aversions among participants, Table S4: Proportions of vitamin- and mineral-supplement users among participants.
