# Supplementary material for: Trimester-Specific Assessment of Diet Quality in a Sample of Canadian Pregnant Women
Source: Int J Environ Res Public Health. 2019 Jan 24;16(3):311. doi: 10.3390/ijerph16030311 (PMC6388152; doi:10.3390/ijerph16030311)
Supplement: Supplementary file 1 [file ijerph-16-00311-s001.pdf]

International Journal of
Environmental Research and Public Health
MDPI

Table S1. Components of Canadian adaptation of Healthy Eating Index, range of scores and scoring criteria.

Component	Maximum Points	Scoring criteria (daily servings) *	
		Minimum score	Maximum score
**Adequacy** †	**60**		
Total vegetables and fruits	10	0	4 to 10 servings
Whole fruits	5	0	0.8 to 2.1 servings
Dark green and orange vegetables	5	0	0.8 to 2.1 servings
Total grain products	5	0	3 to 8 servings
Whole grains	5	0	1.5 to 4 servings
Milk and alternatives	10	0	2 to 4 servings
Meat and alternatives	10	0	1 to 3 servings
Unsaturated fats	10	0	30 to 45 grams/day §
**Moderation** ‡	**40**		
Saturated fats	10	≥15% of total energy intake	≤7% of total energy intake
Sodium	10	twice the UL (4600 mg)	AI or less (≤1500 mg)
Other foods	20	≥40% of total energy intake	≤5% of total energy intake

Adapted from Garriguet D. (2009); UL, Upper Intake limit; AI, Adequate intake. * according to age and sex, as specified in Canada's Food Guide. † for adequacy components, 0 points for minimum intake or less, 5, 10 or 20 for maximum intake or more, and proportional for amounts between minimum and maximum. ‡ for moderation components, 10 or 20 points for minimum intake or less, 0 points for maximum intake or more, and proportional for amounts between minimum and maximum. § Refers only to the amount of unsaturated fats added directly to food during cooking or through salad dressings and does not represents total dietary intake of unsaturated fats.

International Journal of
Environmental Research and Public Health

MDPI

Table S2. Frequency of nausea and vomiting among participants.

	N (%)		
	1st trimester	2nd trimester	3rd trimester
Experienced nausea	70 (88.6)	26 (32.9)	16 (20.3)
>2 times/day	37 (46.8)	3 (3.8)	0
1 time/day	10 (12.7)	1 (1.3)	5 (6.3)
3 to 6 times/week	10 (12.7)	6 (7.6)	1 (1.3)
1 to 2 times/week	2 (2.5)	6 (7.6)	2 (2.5)
Occasionally	10 (12.7)	4 (5.1)	4 (5.1)
Rarely	1 (1.3)	6 (7.6)	4 (5.1)
Experienced vomiting	25 (31.7)	15 (19.0)	2 (2.5)
>2 times/day	2 (2.5)	1 (1.3)	0
1 time/day	4 (5.1)	0	0
3 to 6 times/week	1 (1.3)	1 (1.3)	0
1 to 2 times/week	2 (2.5)	4 (5.1)	0
Occasionally	10 (12.7)	3 (3.8)	1 (1.3)
Rarely	6 (7.6)	6 (7.6)	1 (1.3)

International Journal of Environmental Research and Public Health

MDPI

Table S3. Food cravings and aversions among participants.

		N (%)		
	**Foods included in each category**	**1st trimester**	**2nd trimester**	**3rd trimester**
Number of women who reported cravings		44 (55.7)	33 (41.8)	25 (31.7)
Sweet	Chocolate, sweets, ice cream, soft drinks, desserts	23 (29.1)	22 (27.8)	20 (25.3)
Fruit	Fruit, fruit juice	11 (13.9)	13 (16.5)	8 (10.1)
Dairy	Milk, yoghurt, cheese, cream	17 (21.5)	5 (6.3)	5 (6.3)
Proteins	Meat, poultry, fish, eggs	6 (7.6)	3 (3.8)	4 (5.1)
Carbohydrates	Cereal, bread, pasta, potatoes	13 (16.5)	4 (5.1)	2 (2.5)
Salty snacks	Crisps, hummus, nuts, olives, condiments	17 (21.5)	5 (6.3)	5 (6.3)
Pickles	Pickles	4 (5.1)	0	0
Fast food	Pizza, burgers, fries, sushi, poutine	14 (17.7)	5 (6.3)	4 (5.1)
Vegetables	Vegetables, salads, soups	8 (10.1)	5 (6.3)	2 (2.5)
Number of women who reported aversions		50 (63.3)	28 (35.4)	17 (21.5)
Sweet	Chocolate, sweets, ice cream, soft drinks, desserts	6 (7.6)	0	1 (1.3)
Fruit	Fruit, fruit juice	0	0	1 (1.3)
Dairy	Milk, yoghurt, cheese, cream	3 (3.8)	0	0
Proteins	Meat, poultry, fish, eggs	33 (41.8)	22 (27.8)	14 (17.7)
Carbohydrates	Cereal, bread, pasta, potatoes	2 (2.5)	0	0
Salty snacks	Crisps, hummus, nuts, olives, condiments	10 (12.7)	4 (5.1)	2 (2.5)
Coffee and alcohol	Coffee, beer, wine, liquor	9 (11.4)	4 (5.1)	3 (3.8)
Fast food	Pizza, burgers, fries, sushi, poutine	3 (3.8)	0	2 (2.5)
Vegetables	Vegetables, salads, soups	14 (17.7)	3 (3.8)	2 (2.5)

International Journal of
*Environmental Research
and Public Health*

MDPI

Table S4. Proportions of vitamin- and mineral-supplement users among participants.

	Proportion (%)		
	1st trimester	2nd trimester	3rd trimester
≥ 1 supplement (all types)	86.1	84.8	78.5
Number of supplements (all types) taken during pregnancy			
0	13.9	15.2	21.5
1	68.4	74.7	64.6
2	10.1	6.3	11.4
3 (Maximum)	7.6	3.8	2.5
Type of supplements most commonly taken			
Multivitamins	74.7	77.2	70.9
Folic acid supplement	16.5	7.6	5.1
Vitamin D supplement	7.6	5.1	3.8
Iron supplement	1.3	0	5.1
Omega-3 supplement (mostly EPA-DHA)	5.1	3.8	5.1
